# Capacity Building in Global Mental Health Research

**DOI:** 10.3109/10673229.2012.649117

**Published:** 2012-02-15

**Authors:** Graham Thornicroft, Sara Cooper, Tine Van Bortel, Ritsuko Kakuma, Crick Lund

**Affiliations:** 1 Health Service and Population Research Department, Institute of Psychiatry, King's College London; 2 Centre for Public Mental Health, Department of Psychiatry and Mental Health, University of Cape Town, South Africa; 3 Centre for International Mental Health, Melbourne School of Population Health, University of Melbourne

**Keywords:** capacity building, global mental health, research

## Abstract

Research-generated information about mental disorders is crucial in order to establish the health needs in a given setting, to propose culturally apt and cost-effective individual and collective interventions, to investigate their implementation, and to explore the obstacles that prevent recommended strategies from being implemented. Yet the capacity to undertake such research in low- and middle-income countries is extremely limited. This article describes two methods that have proved successful in strengthening, or that have the potential to strengthen, mental health research capacity in low-resource settings. We identify the central challenges to be faced, review current programs offering training and mentorship, and summarize the key lessons learned. A structured approach is proposed for the career development of research staff at every career stage, to be accompanied by performance monitoring and support. A case example from the Mental Health and Poverty Project in sub-Saharan Africa illustrates how this approach can be put into practice—in particular, by focusing upon training in core transferrable research skills. (harv rev psychiatry 2012;20:13–24.)

Most people in the world who have mental illnesses receive no effective treatment.^1^,^2^ At the national level, the proportion of such people receiving health care interventions is no better than 27% or 30.5% in Europe^3^,^4^ and the United States,^5^ respectively, and has even been documented to be as low as 2% per year in Nigeria.^6^–^9^ Mental health issues are often neglected in low- and middle-income countries (LAMICs) due to the low priority given to mental health on the public health agenda.^10^ This low priority continues despite the available evidence that the global burden of mental illnesses is increasing^11^ and that mental and physical illnesses are interconnected.

In the last decade, there has been increasing international recognition of the importance of addressing mental health on a global scale. The World Health Organization (WHO) dedicated its *World Health Report 2001* to mental health and called upon governments to make mental health a priority and to allocate resources to develop and implement policies to address this problem.^11^ The report's ten recommendations led to the development of the Essential Package for Mental Health Policy, Plans and Services, which includes 14 modules to assist policymakers and planners in implementing the recommendations. The landmark *Lancet* series on global mental health in September 2007 provided a comprehensive evidence base that called for action to scale up services for mental disorders worldwide, especially in LAMICs. This call emphasized evidence-based, culturally appropriate, cost-effective, feasible care, and in the wake of the *Lancet* series, the “treatment gap” for mental disorders has come to be increasingly appreciated worldwide.^12^,^13^

Recognizing the importance of this challenge, the WHO's Department of Mental Health and Substance Abuse launched the Mental Health Gap Action Programme (mh-GAP) in 2008.^14^ The first major product of this program is the Intervention Guide (mhGAP-IG) for delivering mental health interventions by general health practitioners, largely at the primary health care level.^15^ This guide is instructive as it was based both upon the limited evidence from LAMICs and upon evidence from high-income settings, filtered in relation to feasibility and acceptability to people with mental illnesses and their families.^16^

Research-generated information is crucial in order to establish the health needs in a given setting,^17^ to propose culturally apt and cost-effective individual and collective interventions,^18^ to investigate their implementation,^19^ and to explore the obstacles that prevent recommended strategies from being implemented.^10^ The difference between the research information needed to develop the best possible services in a given setting and what is currently available can be referred to as the “research gap.”

In order to reduce this gap in LAMICs, WHO launched a program called *Research for Change*, which is supported by stakeholders that include policy advisers and program planners from LAMICs, representatives of national research institutes and research councils, international research organizations, editors of scientific journals, and funding agencies and donors.^20^ Against that background, the purpose of this article is to review some of the challenges of building mental health research capacity in LAMICs, along with some of the principles and strategies that can be adopted to address these challenges.

## CHALLENGES OF BUILDING MENTAL HEALTH RESEARCH CAPACITY IN LAMICs

In LAMICs, evidence for the delivery of mental health care has been scarce. Five to ten years ago, only 10% of the world's health research addressed the 90% of the global population living in LAMICs,^21^,^22^ and only 3%–6% of the mental health research in high-impact medical journals was from LAMICs.^23^–^26^ The sparse evidence available on effective interventions for mental disorders in LAMICs seems to be limited to local settings and has not been scaled up to systematic evaluations of regional populations.^27^ Since research will thus play a critical role in improving health, equity, and development,^28^,^29^ mental health research evidence is urgently needed to inform policymakers and planners in developing the most appropriate mental health care package in resource-poor settings.^30^

To increase funds for research will be of limited benefit, however, without an established capacity to conduct and apply high-quality research. A key challenge in many LAMICs is the weak capacity for such research—in particular, research directly relevant to the most pressing mental health challenges in those settings.^21^,^31^ We include in our definition of research capacity the ability to conduct, manage, disseminate, and apply research in policy and practice.

Within the context of mental health, the Global Forum for Health Research, in collaboration with WHO, mapped research capacity in LAMICs in 2004 with the aim of raising awareness of the need to strengthen research capacity regarding mental health.^32^ Evaluation of research outputs from 114 LAMICs found that 58% of countries contributed fewer than five articles each to the indexed literature on mental health between 1993 and 2003 and that countries such as Argentina, Brazil, China, India, the Republic of Korea, and South Africa were significantly more active. Over half of the respondents (mental health researchers and 290 “stakeholders”) had not received formal training in epidemiology, public health, or basic sciences. Financial support for training was low, and technical support to carry out research, including access to the relevant literature, was also limited. As reported by researchers, the three leading challenges for mental health research were lack of funds, trained staff, and time (the WHO's *Mental Health Atlas 2011* [http://www.who.int/mental_health/publications/mental_health_atlas_2011/en/index.html] summarizes the current data on staffing levels). Lack of research culture and lack of collaborators were also considered important challenges. These problems are not specific to mental health research. In many African academic institutions, for instance, research in basic sciences, applied sciences, and humanities has been poor due to insufficient remuneration, heavy teaching loads, lack of mentorship, and inadequate infrastructure. Furthermore, in many LAMIC settings, since institutional review boards are lacking or dysfunctional, the ethical standards for research may be inadequate.

This mapping project highlights the gaps in capacity for mental health research at the individual, institutional, organizational, and national levels. It demonstrates the urgent need for initiatives to strengthen research capacity, including skills in epidemiological or public health research methods, knowledge translation and exchange, leadership, mentorship, and advocacy. Increasing such capacity at all levels will yield a greater impact and provide a stronger infrastructure to support mental health research.^33^ At present, however, LAMICs give low priority to mental health, in general, and to mental health research, in particular. The Commission on Health Research for Development estimated that in 1990, less than 10% of global health research resources were being used for the health problems of LAMICs, which accounted for over 90% of the world's health problems—termed the *10/90 gap*.^34^

An overview of mental health research was conducted in 30 Latin American and Caribbean countries in order to understand the challenges in reducing the 10/90 gap.^35^ Most countries allocated less than 1% of their gross domestic product to research and human development, and less than 1% of their health budgets to mental health,^11^ with the consequence that mental health research publications from LAMICs comprise a small proportion of the world's total research output on mental health. Mental health–related publications amount to less than 4% of the global health literature, yet mental disorders account for 13% of the global burden of disease.^21^ Mental health research has been even more neglected in LAMICs, where mental disorders account for an important fraction of the burden of disease. In recent years, however, studies in Latin American and Caribbean countries indicate that the situation is improving, with increases in the number of scientific publications in journals indexed by MEDLINE and ISI's Web of Knowledge, in the number of master's and doctoral students, and in research funding.^36^–^40^

Research in mental health research must be understood as part of the larger effort to conduct research in the field of health care generally. In this broader context, LAMICs must not only increase their research funding but also build research capacity (e.g., trained researchers, technical support, peer networks, cooperative endeavors, migration of researchers) and create a viable research environment (e.g., research culture, availability of time, incentives, administrative support).^31^,^41^ Another key problem for some LAMICs is the increasing “brain drain” (especially for junior researchers), which can be addressed only through national policies to ensure support for research and promotion of research careers. At present, however, LAMICs' research budgets do not allocate funds for the salaries of researchers, many of whom work as volunteers. To help halt the brain drain and to boost research efforts, it would also be helpful to hold regional conferences where, among other things, key strategies for improving research capacity could be discussed. Such strategies include training mental health professionals in research methods and scientific writing; making mental health research attractive to young researchers; promoting strategies for acquiring research grants and for developing and sustaining researchers' careers; increasing the level of networking among research teams; enhancing research dissemination; and fostering dialogue between research teams and policymakers.

Looking specifically at mental health research in LAMICs, the paucity of such research has been amply documented,^20^,^21^,^23^,^42^,^43^ and mental health research outputs in Africa are especially thin; between 1993 and 2003, African countries contributed only 12.5% of the indexed mental health literature.^32^ The low mental health research output by the majority of LAMICs can be understood in relation to factors such as the shortage of mental health professionals, graduate programs, and mental health professionals involved in educational and research activities. Other underlying reasons include poor funding for mental health research; limited numbers of trained mental health research personnel; a dearth of infrastructural support and research networks; and the absence of institutions with a research culture.^44^,^45^

There are also other obstacles to increasing the number of publications from LAMICs in peer-reviewed journals. Anecdotal evidence suggests that many researchers assume that their papers will not be accepted in high-impact journals and hence aim for lower-impact or more localized (often non-indexed) journals. Others do not even bother trying to publish their results. Problems such as English-language proficiency and the unaffordability of submitting to open-access journals have the effect of even further discouraging academic writing.^46^ Journal editors may actually be less likely to publish manuscripts from LAMICs.^47^,^48^ The number of theses that never get published is likely to be significant, with the consequence that potentially useful research may be neglected. Taking all these factors into account, especially given funding constraints, the weak research culture, and difficulties finding collaborators, researchers from LAMICs often have little incentive to write academic papers. Given the high demand for mental health research in these countries, however, these obstacles must somehow be overcome.

## STRENGTHENING RESEARCH CAPACITY IN LAMICs

To begin to address the above challenges, the highest priority areas for mental health research have been identified as health policy and systems research, delivery of existing cost-effective interventions in low-resource contexts, and epidemiological research on child and adolescent mental health and substance abuse.^49^,^50^

Many funding agencies are now responding to the need for significant investment in building research capacity. For example, the UK Department for International Development Research Programme Consortium requires projects to allocate a particular amount of time and resources to increase research capacity, support career development of research officers, and disseminate findings.^51^ The WHO Alliance for Health Policy and Systems Research focuses on enhancing LAMIC capacity for applying research findings in policymaking (http://www.who.int/alliance-hpsr/projects/strategies_capacity/en/index.html). North-South research partnerships have played key roles in strengthening capacity, and South-South partnerships are becoming increasingly important.^52^ Recently, the Wellcome Trust launched a £30 million initiative for over 50 African institutions to lead international consortia to strengthen research capacity on that continent, and the Indian government and Wellcome Trust jointly committed £80 million over five years to strengthen biomedical science research in India (http://www.wellcome.ac.uk).

The World Psychiatric Association has provided support to identify steps needed to get non-indexed journals indexed. Training programs such as the Fogarty Training Program in International Mental Health (sponsored by the U.S. National Institutes of Health for work conducted in China), a master's of science program in psychiatry for developing-country students (sponsored by the United Kingdom), the McGill University summer program in transcultural psychiatry, and the Harvard Medical School/University of Melbourne International Mental Health Leadership Program have been in place for many years. New programs to increase mental health research capacity and to scale up mental health services are increasing (see text box).

During the past decade, greater investment in training researchers has boosted the development of mental health research in LAMICs. Different countries and institutions have different needs and capacity-strengthening priorities. The country-specific needs and institutional priorities may result in slightly different methods of training and different activities being undertaken in each country. Among the major challenges presented by these collaborations are the need to integrate the different research interests and priorities of the various partners and the need to overcome language barriers, especially for qualitative projects and publications.

**Existing Initiatives/Programs to Strengthen Research Capacity in Mental Health**

International Mental Health Leadership Program (Melbourne, Australia; 2001 to present). http://www.cimh.unimelb.edu.au/pdp/imhlp

International Mental Health Research—Methods & Applications, London School of Hygiene and Tropical Medicine/Institute of Psychiatry http://www.lshtm.ac.uk/prospectus/short/simh.html

Leadership programme (Goa, India; 2008 to present) http://www.sangath.com/sangath/files/otherpdfs/leader-shipJn_mental_health_course_announcement_for_registration.pdf

International Master on Mental Health Policy and Services (Lisbon, Portugal) http://www.fcm.unl.pt/gepg/index.php?option=com_content&task=view&id=400&Itemid=420

Interdisciplinary Postgraduate Training in Mental Health Policy and Economics Research (Venice, Italy) http://www.icmpe.org/testl/training/index.htm

Zambian Forum for Health Research (ZAMFOHR), Fellowship program in knowledge translation (Lusaka, Zambia), and Mental Health Research-to-Action Group http://www.zamfohr.org

International Diploma in Mental Health Law and Human Rights (Indian Law Society/WHO) http://www.mentalhealthlaw.in

Canadian Coalition for Global Health Research, Summer Institute and Global Mental Health Research Group http://www.ccghr.ca

University of Cape Town–Stellenbosch University Joint Postgraduate Diploma and master's degree (MPhil) in Public Mental Health (in development; proposed launch date 2012) http://www.cpmh.org.za

### Case Study: Building Research Capacity in Sub-Saharan Africa in the Mental Health and Poverty Project

Weak mental health research capacity, infrastructure, and outputs in Africa are a major source of concern. Increasing mental health research related to Africa has been identified as essential to improving the mental health situation there, to reducing the burden of common and disabling disorders, and to facilitating economic growth, development, and equity, including the fight against poverty.^13^,^34^ Research-generated information helps to identify local needs, select local priorities, propose cost-effective interventions, monitor their implementation, and evaluate their effectiveness. The massive imbalance between health research in LAMICs and high-income countries (HICs) means that mental health programs and policies in Africa, insofar as they exist at all, are either not evidence based or are based primarily on evidence from HICs, which may be disconnected from local needs, priorities, and realities. Few policies, programs, and interventions in Africa are therefore based on information derived from research that is structurally, culturally, financially, and contextually valid and relevant to Africa.^4^ For these reasons there is a pressing need to improve and strengthen individual and institutional mental health research capacity and infrastructure in Africa.

The UK Department for International Development-funded Mental Health and Poverty Project^51^ (MHaPP) undertook evaluations of mental health care systems in four sub-Saharan countries (Ghana, South Africa, Uganda, and Zambia), provided interventions to assist in developing and implementing mental health policies in those countries, and evaluated policy implementation during 2005–10 in collaboration with WHO and the University of Leeds. In particular, MHaPP investigated strategies for making mental health care accessible to poor communities through primary health care and nonhealth sectors.^53^–^57^

One of the four objectives of MHaPP was capacity development, which was integrated into the overall MHaPP research program. All MHaPP partners went through a process of identifying their own needs for developing research capacity and attended a variety of training workshops. Supervision/mentoring processes and postgraduate study were also included when necessary and possible. These capacity-development activities were yoked to specific research activities, depending on the stage of the overall consortium. For example, as part of the preparation for the first phase of fieldwork, research officers were provided with training in semistructured interviews, instrument design, and qualitative data collection. Once data were available, further training was provided in qualitative data analysis, academic writing, and publication of papers in peer-reviewed academic journals. Specific efforts were made to support all research officers to publish as first authors and to increase publication opportunities such as special issues dedicated to MHaPP findings (e.g., in the *African Journal of Psychiatry and International Review of Psychiatry*).

### Implications for Other Capacity-Development Initiatives

Numerous lessons emerged from the MHaPP capacity-development experience,^53^,^56^ which may be relevant to other such initiatives for mental health research in LAMICs:

- give training to both senior and junior investigators, as senior staff may have gaps in their earlier research training or need updating on recent research design or methods of data analysis
- use diverse methods for intercountry communication, including email, teleconferences, Skype, and face-to-face meetings
- stimulate site visits between research centers
- identify a lead person per site who is responsible for local-capacity development and for liaising with other network members
- adopt train-the-trainer approaches, so that local individuals can provide ongoing training and support to other team members (e.g., when staff turnover is high)
- implement an online journal club to discuss and critique key articles
- identify specific strengths of each partner that can be shared with the rest of the collaborative group
- build into the capacity-development activities ones that are specific to extending the skills of senior, midgrade, and junior investigators
- organize opportunities for junior staff to practice key skills (e.g., oral presentations, posters, and grant applications) live with feedback from peers and senior staff

## PERFORMANCE MONITORING AND MANAGEMENT

It is now increasingly common in HICs to explicitly monitor and manage the academic performance of research staff.^58^ The use of such metrics can also be applied to improving the capacity and capability of researchers in LAMICs.

Expected outcomes for capacity development, for example, could include authorship of peer-reviewed publications; researchers' involvement in grant applications for multiplier funding, especially fellowships; multiplier funds generated for mental health research; researchers trained in mental health research methods; linkages with other regional training programs for building health research capacity; support for mental health by public health institutions; establishing mental health research courses in each country; establishing new collaborations between network partners; and the use of research by decision makers and other stakeholders.

Given the increased focus on, and funding for, the development of health research capacity, the monitoring and evaluation of that capacity have received increased attention. A monitoring-and-evaluation framework for health research capacity development was developed by ESSENCE for Health Research, a network of funding agencies working together to harmonize their programs and monitoring-and-evaluation strategies. The framework comprises a set of indicators of activities, outputs, and outcomes at individual, organizational, and national research system levels (see http://apps.who.int/tdr/svc/partnerships/initiatives/essence/). Another such framework was developed by the Canadian Academy of Health Sciences; it further outlines specific indicators for evaluating the impact of investing in health research.

Appropriate indicators of successful capacity-strengthening programs must be explored further. The ESSENCE framework, like many other similar frameworks, has yet to be validated in LAMIC contexts, and neither has the Canadian framework, which was primarily targeted for high-income settings. Indicators such the volume of academic output, involvement with grant applications, training programs, available resources, and number of partnerships in LAMICs may reflect only a component of the available research capacity. First, in addition to just numbers, measurements must capture various shifts in roles, such as the increased number of publications as first author, increased number of grant applications as principal investigator, and increased participation in teaching, supervision, and mentorship.

Second, if researchers are conducting research “for change,” academic papers are not necessarily the most effective method for disseminating research results. The literature on knowledge transfer and exchange, while fully recognizing the importance of academic papers, emphasizes the need for multiple dissemination strategies such as policy briefs, reports and presentations at conferences, and key meetings with decision makers.

Partnership programs for training researchers in LAMICs contribute significantly toward strengthening both sustainable individual research skills and institutional capacity to support research and research careers in Southern partner institutions.^59^,^60^ Strategies to be used include training programs, postgraduate programs, workshops, joint projects, joint publications, mentorship, staff exchanges, and long-term secondments. To safeguard against ineffective and often inequitable partnerships and to monitor the impact of the partnerships in place, some sort of evaluation process is required; various assessment tools are available for this purpose.

**Transferable Research Skills**

- research design
- methodologies (qualitative and quantitative) and systematic reviews^61^
- methods of data analysis
- conducting a situation analysis and needs assess ment of mental health services in the population
- accessing scientific literature and databases (e.g., see http://www.who.int/hinari/en/)
- evaluating the implementation of services
- organizational management such as growing research teams
- managing conflict in research groups and supervising staff in research teams
- leadership and building alliances
- fundraising
- advocacy and donor-relationship skills
- disseminating research findings in various formats
- writing peer-reviewed papers
- developing, reviewing, and critiquing grant proposals
- making presentations
- chairing and recording meetings
- increasing contributions to indexed journals
- using of online peer group networking

## STAGES OF RESEARCH CAPACITY DEVELOPMENT

It is possible to envision the development of research capacity in LAMIC sites as a closely interconnected sequence of steps. Colleagues need to be identified and then prepared for involvement in mental health research (in all its stages)—the first step in developing a cadre of competent, dedicated researchers to lead the field. At each stage, and linked to this structure, we envisage various activities that will impart important, transferable skills, graded according to the level of expertise and seniority required (see text box).

### 1. Introduction to Research

At this stage, nonresearcher colleagues—for example, clinical practitioners or health policy staff—are invited to attend introductory sessions about research and such matters as evidence-based treatment guidelines (e.g., mhGAP-IG). Communicating in nontechnical language, the focus is on practical information that is directly usable, emphasizing “what works.” Transferable skills include awareness of evidence-based practice paradigms and basic familiarity with accessible sources of relevant evidence.

### 2. Initial Familiarization with the Work of Research Teams

The next stage involves such activities as attending special sessions, potentially full-day events, where interesting new findings are presented, while also emphasizing the rewards of discovery-led research. Transferable skills include understanding both the process of developing research questions and how that process leads, in turn, to the discovery of clinically important information.

### 3. Attendance at Short Courses on Specific Research Themes

People who are identified as taking an interest in research should be invited to attend short courses (e.g., lasting 2–5 days), with the goal of developing their capacity for intervention research in primary health care and community settings. These courses can be customized to the needs of specific target audiences and need to be followed up with monitoring, support, and supervision of the trainees. Methods to provide more extensive learning opportunities should also be explored, such as developing IT/phone/video infrastructure in Southern partner countries to facilitate distance-based learning. Transferable skills include research design, methodology (qualitative and quantitative), and analysis; conducting situation analyses, along with needs assessment, regarding mental health services in the local population; setting appropriate targets for service development; evaluating the implementation of services; organizational management, such as growing research teams in complex organizations, managing conflict in research groups, and supervising staff in research teams; leadership; building alliances; fundraising, advocacy, and donor relationships; disseminating research findings in various formats, including writing peer-reviewed papers; developing, reviewing, and critiquing grant proposals; making presentations; chairing and recording meetings; methods to increase the citation counts of scientific publications; and contact with research colleagues via social-networking Internet sites.

### 4. Master's-Level Programs

The master's-level programs, offered by the Northern partner institutions, are likely to include the following elements:

- understanding of the scientific and ethical principles common to all mental health research disciplines
- knowledge of, and skills in, social, epidemiological, quantitative, or qualitative research methodologies
- developing the ability to critically appraise mental health research and apply its findings to specific mental health problems and settings
- learning how to formulate answerable research questions, select appropriate study designs, and implement mental health research
- experience of key research skills such as collaborating within a larger research consortium, conducting a structured or systematic literature review, or writing a research protocol
- understanding of strategies for disseminating the results of research
- developing the ability to write and publish scientific papers
- conducting rapid research assessment and appraisals

A wider range of transferable skills is pertinent at the master's stage, including: writing a conference abstract; presenting an oral paper at a conference; presenting a poster at a conference; drafting a scientific paper or letter; working in a group to edit a scientific paper; deciding on the paper title; appreciating the importance of the paper abstract; submitting a scientific paper to a journal (including careful attention to the cover letter itself); critically appraising a scientific paper; conducting a systematic or structured review; understanding scientific metrics, including impact factor, citation index, and Hirsch index; formulating answerable research questions; specifying research hypotheses; deciding between options for the research design of studies; understanding the mechanics and construction of research grant applications; and having a detailed working knowledge of the locally applicable research ethics framework and procedures.

### 5. Predoctoral Preparatory Fellowship and Doctoral Fellowships

To suitably qualified staff, a critical career stage is to gain access to a PhD program—for example, through a specifically dedicated fellowship. Immediately transferable skills include advanced skills in research design, ethical applications, and research governance; and advanced-level skills in the transferrable skills given under stage 4.

### 6. Postdoctoral Research Positions and Opportunities

Opportunities will be given to postdoctoral researchers in hub countries to lead small- to medium-sized projects, to be primary investigators on the related grant applications, and to be co-applicants on grants for larger projects. Transferable skills include learning to recruit, supervise, appraise, and offer career support to junior research staff; project-management and budget-management skills; identifying problems in recruiting study participants, along with appropriate remedies; liaison with external stakeholders, including funders and advisory and steering boards; establishing publication protocols and plans; identifying and dealing with conflict between research partners; designing and implementing a dissemination strategy for research results; creating and maintaining effective research partnerships and networks; and skills for chairing research meetings and recording their proceedings.

Graduate programs tend to foster research culture and quality; as such, they typically have a positive impact on the number of publications and the dissemination of research findings. The development of rigorous systems for graduate-program evaluation is a crucial factor in strengthening institutions in LAMICs, as is apparent from Razouk and colleagues' study of Brazil.^37^ Over a five-year period, graduate programs trained about 50 doctoral students per year. Predictably, the number of MEDLINE and ISI Web of Science publications from the country doubled during this period. In order to achieve this change, Brazil established the so-called sandwich scholarship, a fellowship scheme that covers, among other things, expenses for students to spend one year abroad as part of their training.^38^

Postgraduate training, frequent exchanges, mentorships, and postgraduate-related periods spent at the Northern partner institution all facilitate familiarity with host institution staff, their research, and teaching procedures, and promote interaction with the international research community. These activities can lead, in turn, to a greater feeling of partnership between North-South institutions (also combating the sense that Southern countries are simply providing research material for Northern authors). More generally, studies have shown that postgraduate training contributes significantly to enhancing the advanced research capacities of LAMICs. In this context, it is imperative to invest in LAMICs' institutional capacity to teach research skills; training should take place in LAMIC settings and should focus on equipping people with the skills to become teachers or trainers themselves. An emphasis on training persons to be trainers helps to encourage local ownership and ensure the sustainability of local capacity, especially in relation to frequent staff migration (a reality in many LAMICs).

The Toronto Addis Ababa Psychiatry Project for the postgraduate psychiatry training program in Ethiopia—a collaboration between Addis Ababa University and University of Toronto, http://www.utoronto.ca/ethiopia/fac__res_info.htm—is a good example of this training trainers approach.^62^ A further considerable challenge is the lack of access to journal publications. An alternative to online access is to use CD versions of journal contents and abstracts, which can greatly enhance access to the latest scientific findings. At the University Teaching Hospital in Lusaka, Zambia, where the Internet connection is still unreliable, students' access to online journals is facilitated by downloading materials onto university computer hard drives (personal communication, Dean of School of Medicine, University of Zambia, February 2008).

### ADDITIONAL MEANS OF PROMOTING RESEARCH

#### Long-Term Secondments

Another influential factor in both individual and institutional capacity building for research in LAMICs is the availability of full-time, long-term secondments. Having a dedicated person in place facilitates onsite guidance and support on a wide range of research-related issues. Both partner institutions often recognize long-term secondment staff as a pivotal investment in building research capacity and ensuring that the work delivered by the Southern institution meets international standards. The use of secondments has strengthened relationships between North-South partner institutions and led to increases in research projects, joint activities, and joint publications.

### Joint Projects

Joint projects can be a core component of collaborations. North and South institutions can implement joint projects on topics of mutual interest and jointly disseminate the resulting knowledge. Partnerships in themselves might not necessarily provide research funds, but the involvement of LAMIC institutions in joint proposals can improve their chances of receiving funding. Some of these joint projects can be financially large and contribute significantly to institutional income. The methods used to achieve and enhance collaborative developments and implementation of projects include frequent email contact, face-to-face meetings, and mutual visits and workshops, all of which assist the joint assessment of progress, discussion of results, and report writing.

### Joint Publications

The proportion of peer-reviewed articles with Southern first authors is still too low. In this context, partnerships and project collaborations effectively increase the number of papers produced and consequently provide increased opportunities for LAMIC authors to serve as first authors. Adequate support to junior staff interested in first-authoring papers remains a challenge for all partners, but explicit efforts can be made through writing workshops and mentorship.

Past studies have shown that formal partnerships have resulted in significant strengthening of individual research skills and in moderate institutionalized strengthening in LAMIC institutions.^59^ But partnerships also present significant benefits for HICs. The majority of HICs face shortages of mental health specialists and inequitable access to services across communities, and are therefore seeking strategies to provide cost-effective and equitable care. Given that HICs are becoming more culturally diverse, international research collaborations provide opportunities for HIC researchers to gain knowledge on how to better provide culturally appropriate services in their own countries. The publications arising from joint projects can also increase international recognition of the value of the underlying research.

Compared to other health fields and mental health researchers in HICs, mental health researchers in LAMICs require a broader range of skills to achieve the same goals. Not only do they need to acquire the same basic research methodology and communication skills as other researchers, they must also have strong advocacy, leadership, and mentorship skills to increase research demand and establish a sustainable research network—not to mention the practical requirements of office space, Internet access, and other essential nonsalary costs. There are also certain challenges associated with publishing research about LAMICs in more broadly based journals.

Existing evidence and personal accounts from researchers worldwide suggest that a pragmatic approach to building research capacity must be implemented in LAMIC settings. Given the significant shortages in human health resources, coupled with the lack of funding, lack of time, and other challenges identified by the Global Forum for Health Research,^32^ strategies for strengthening research capacity must also simultaneously contribute toward combating these deficiencies. In the context of international partnerships, efforts to build research capacity through systematic reviews, critical appraisals of past and present research and research designs, preparing grant proposals, and writing academic papers and other outputs can serve to generate practical outcomes. These activities provide opportunities to learn about the broad range of available evidence and how to find it; to better understand research methodology; and to gain skills in interpreting research results properly in view of the actual quality of any particular study. Likewise, the process of preparing grant proposals can be understood as an opportunity to strengthen research capacity. Proposals need to include thorough literature reviews, describe and justify the study's methodology, and articulate with a clear knowledge-to-action plan how the study will influence policy and practice, and how it will benefit local populations clinically and economically.

### Mentorship

Any plan to build research capacity needs to include an explicit component for mentorship, leadership, and advocacy. Partners with greater research experience and expertise must commit to mentoring their less-experienced partners in all of the ways discussed above, and with the understanding that the ultimate goal is for the LAMICs to develop their own, self-sustaining research capacity. Rather than only identifying gaps/weaknesses and working to address them, an effort should be made to identify the strengths of each person and to provide opportunities for those strengths to be shared with others. Southern researchers should also be explicitly reminded that they have something to contribute that will help improve mental health care in Northern countries.^63^

In an area such as global mental health, where awareness and resources are limited, more intentional efforts must also be made in supporting the “leaders” for mental health research in their own settings so that they can better advocate for and champion mental health.

### Integration of Mental Health into Public Health Training

Funding bodies are increasingly allocating resources toward strengthening health research capacity in developing countries. Many of these efforts are specifically aimed at developing MSc and PhD programs in epidemiology, biostatistics, and public health, and include the scholarships required to recruit students. Other efforts are focused on strengthening national health systems and national health research systems. As mentioned earlier, large-scale international consortia to strengthen health research capacity are currently in place across Africa and India. Integrating *mental* health into existing or upcoming programs in epidemiology or public health and into initiatives to strengthen national health research systems would not only be cost-effective but increase the capacity of health researchers more broadly to understand the importance of mental health and to incorporate mental health issues in their own work.

## CONCLUSION

The need to strengthen sustainable research capacity in the South has been long appreciated.^64^ In this article we have described practical ways in which such strengthening can be planned and implemented. As in any other area of human services, expertise accumulates slowly; a long-term commitment and sustained efforts are required. The recent surge of interest in global mental health^14^ has included the 2007 *Lancet* series on global mental health, the WHO mhGAP Implementation Guide, the World Psychiatric Association Task Force on developing mental health services in low-income settings,^65^,^66^ site-specific and multinational studies (including randomized, controlled trials)^67^–^69^ in low-income settings,^70^–^74^ and the new, recently launched 2011 *Lancet* series on global mental health. If these initiatives are to be sustained in the long term, it is vital that individual, institutional, and system research capacity be built in LAMICs. The time is right to invest with renewed vigor in mental health research capacity for global mental health.

We also recommend that such research-capacity activities be based upon explicit principles to guide action. One example is the UK Department for International Development's “Ten Steps to Good Capacity Building” (part of the first-listed initiatives listed in the text box on the next page). Other existing initiatives to strengthen health research capacity (generally) include the Wellcome Trust African Institutions Initiative, the Health Research Capacity Strengthening Initiative, the WHO Alliance for Health Policy and Systems Research, the Initiative to Strengthen Health Research Capacity in Africa, and the Medical Education Partnership Initiative.

**Existing Initiatives/Programs to Build Health Care Research Capacity**

Department for International Development (UK), Research Programme Consortia. Guidance Note on Capacity Building. http://www.dfid.gov.uk/r4d/PDF/Publications/Guidance-Note_CapacityBuilding.pdf

Wellcome Trust. Platform for Research—African Institutions Initiative. http://www.wellcome.ac.uk/News/2009/Features/WTX055738.htm

Department for International Development (UK), International Development Research Centre (Canada), and Welcome Trust. Health Research Capacity Strengthening Initiative: Kenya and Malawi. http://www.wellcome.ac.uk/stellent/groups/corporatesite/@sf_cross_cutting_activities/documents/web_document/wtx035037.pdf

World Health Organization, Alliance for Health Policy and Systems Research. http://www.who.int/alliance-hpsr/en/

TDR (Special Programme for Research and Training in Tropical Diseases). Initiative to Strengthen Health Research Capacity in Africa. http://apps.who.int/tdr/svc/grants/calls/ishreca

National Institutes of Health (U.S.), Fogarty International Center. Medical Education Partnership Initiative. http://www.fic.nih.gov/Programs/Pages/medical-education-africa.aspx

In conclusion, given that the expropriation of research expertise from LAMICs to high-income settings is a continuing threat to the viability of research capacity in situ in the South, we propose a set of principles to guide capacity development:

- conduct research training largely in low-income settings (at present, for example, much capacity development in Africa is based on a model in which selected African researchers train in overseas settings and perhaps do not return or do develop appropriate skills for training others).
- seek reciprocity in North-South and South-South relationships, and promote a collective culture of transparency
- develop clear output and outcome metrics/indicators for use across projects
- put into practice the principle of subsidiarity—that is, the wider regional level will undertake only what can not be done at the country level
